# Green Synthesized Silver Nanoparticle-Loaded Liposome-Based Nanoarchitectonics for Cancer Management: In Vitro Drug Release Analysis

**DOI:** 10.3390/biomedicines11010217

**Published:** 2023-01-14

**Authors:** Priyanka Jayachandran, Suganya Ilango, Vivekananthan Suseela, Ramalingam Nirmaladevi, Mohammed Rafi Shaik, Mujeeb Khan, Merajuddin Khan, Baji Shaik

**Affiliations:** 1 Department of Biochemistry, Biotechnology and Bioinformatics, Avinashilingam Institute for Home Science and Higher Education for Women, Coimbatore 641043, Tamilnadu, India; 2 Department of Biochemistry, P.S.G College of Arts and Science, Coimbatore 641014, Tamilnadu, India; 3 Department of Chemistry, College of Science, King Saud University, Riyadh 11451, Saudi Arabia; 4 School of Chemical Engineering, Yeungnam University, Gyeongsan 38541, Republic of Korea

**Keywords:** silver nanoparticles, liposomes, dynamic light scattering, drug release, mathematical models, release kinetics, Higuchi model, targeted delivery, cancer cells

## Abstract

Silver nanoparticles act as antitumor agents because of their antiproliferative and apoptosis-inducing properties. The present study aims to develop silver nanoparticle-loaded liposomes for the effective management of cancer. Silver nanoparticle-encapsulated liposomes were prepared using the thin-film hydration method coupled with sonication. The prepared liposomes were characterized by DLS (Dynamic Light Scattering analysis), FESEM (Field Emission Scanning Electron Microscope), and FTIR (Fourier Transform Infrared spectroscopy). The in vitro drug release profile of the silver nanoparticle-loaded liposomes was carried out using the dialysis bag method and the drug release profile was validated using various mathematical models. **A** high encapsulation efficiency of silver nanoparticle-loaded liposome was observed (82.25%). A particle size and polydispersity index of 172.1 nm and 0.381, respectively, and the zeta potential of −21.5 mV were recorded. FESEM analysis revealed spherical-shaped nanoparticles in the size range of 80–97 nm. The in vitro drug release profile of the silver nanoparticle-loaded liposomes was carried out using the dialysis bag method in three different pHs: pH 5.5, pH 6.8, and pH 7.4. A high silver nanoparticle release was observed in pH 5.5 which corresponds to the mature endosomes of tumor cells; 73.32 ± 0.68% nanoparticle was released at 72 h in pH 5.5. Among the various mathematical models analyzed, the Higuchi model was the best-fitted model as there is the highest value of the correlation coefficient which confirms that the drug release follows the diffusion-controlled process. From the Korsmeyer–Peppas model, it was confirmed that the drug release is based on anomalous non-Fickian diffusion. The results indicate that the silver nanoparticle-loaded liposomes can be used as an efficient drug delivery carrier to target cancer cells of various types.

## 1. Introduction

Cancer is an abnormal cell growth that invades other parts of the body and is considered a major cause of death all over the world [1]. In 2019, there were 9.5 million deaths due to cancer and there were 18.1 million new cancer cases [2]. It is estimated that by the year 2040, there will be an increase in new cases and death by up to 40–50%. More than a hundred types of cancers had been recorded so far [3], which are typically categorized based on their origin, for example, blood cancer, lung cancer, and breast cancer, which originate in the blood, lungs, and brain, respectively [4]. Cancer can also be characterized based on the cells that are responsible for their formation such as squamous cells or epithelial cells [5]. These cancer cells can invade other parts of the body through the process called metastasis which results in the formation of secondary tumors [6]. Currently, chemotherapy is the major treatment strategy for most cancer types [7]. These chemotherapy treatments lead to several side effects as they cause damage to both normal and cancer cells [8]. Since there are enormous side effects in chemotherapy, an alternative treatment strategy for cancer with minimal or fewer side effects is the need of the hour [9,10].

Nowadays, silver nanoparticles are increasingly utilized in the treatment of various tumors because of their biosafety, physical properties, and small size [11]. Since they are smaller in size, they can easily penetrate most of the cells and exert targeted drug delivery [12]. There are numerous methods that are available for the synthesis of these nanoparticles, including physical chemical, and biological approaches [13]. Out of these approaches, nanomaterials including silver nanoparticles, obtained from biological techniques typically exhibit higher biocompatibility with human organs and tissues [14]. These methods do not involve hazardous chemical substances and reagents which are toxic and thus can be harmful to both humans and the environment [15]. Particularly, plant extract-based biological methods which involve bioactive secondary metabolites such as flavonoids, alkaloids, saponins, carbohydrates, proteins, and tannins, as natural reducers and/or stabilizers have gained immense popularity [16]. Moreover, these methods are also preferred due to their efficient ability to rapidly produce high-quality nanomaterials at a low cost and in an eco-friendly manner [17]. The major advantage of these nanoparticles is that they produce minimal side effects, and because of the use of plants as a source of primary reagents, nanoparticles obtained from such methods have been widely used in the treatment and diagnosis of cancerous cells [18,19].

So far, different types of plant extracts have been applied to obtain a variety of nanomaterials [20,21]. One such plant is *Tabebuia pallida*, which is often called “Pink trumpet tree”, possesses great potential in the plant-based synthesis of nanomaterial due to the presence of large contents of phenolic and flavonoid compounds [22]. This plant belongs to the family “Bignoniaceae”, which is one of the largest genera that is distributed in South America, Central America, and the western region of India [23]. It is usually grown as an ornamental tree that reaches up to 25–30 m in height. Tabebuia species are mainly used in Ancient folk medicine for treating various diseases including allergies, syphilis, constipation, malaria, prostatitis, cutaneous infections, diabetes, stomach disorders, depression, cancer, irritability, inflammation, poor memory, pain, anxiety, and fungal and bacterial infections [24,25]. *Tabebuia pallida* has been previously used to prepare nanoparticles and is also selected to prepare silver nanoparticles in the current study [26].

The utilization of silver nanoparticles for medicinal purposes is often hampered due to the low solubility of silver-based materials in biological fluids [27]. To subdue these issues, silver nanoparticles are usually conjugated with biologically benevolent entities such as biocompatible organic ligands and biopolymers which enhance the bioavailability of silver-based nano-systems [12]. Notably, bio-conjugated silver nanoparticles not only enhance the solubility of resulting materials by inhibiting agglomeration, but they also offer an oligodynamic effect and enhanced interactions with desired biomolecules [28]. Hence, surface functionalization of nanomaterials with suitable biomolecules in a controlled fashion without disturbing the bulk properties of the parent substance is an effective methodology to obtain biologically effective high-quality silver nanoparticles [29]. Depending upon the requirement, silver nanoparticles are often conjugated with a variety of ligands with diverse functional groups. Particularly, in this regard, tailoring the surface of silver nanoparticles with biologically gentle, hydrophilic functionality is a popular method [30]. There are a large number of naturally occurring biomolecules with different compositions, sizes, and complexity which have been extensively exploited to obtain highly effective, biocompatible, silver-based nano-systems [31]. These ligands include small molecules such as peptides, vitamins, lipids, and larger molecules such as natural polymers including proteins, enzymes, DNA, and RNA [32].

In the case of nanoparticle-based pharmaceuticals, including silver, prolonged biodistribution, and high concentrations of nanomedicines may induce toxicity, due to the inefficient delivery of drugs to targeted cells [33]. Therefore, the development of methods for the effective delivery of low concentrations of nanomedicines to target cells becomes pertinent [34]. One of the effective approaches to achieve this is the encapsulation of nanomedicines in a lipid bilayer. This approach has been already explored in several studies in which the system was found to exhibit improved delivery with promising results [35]. In this regard, liposomes exhibit a variety of novel characteristics that make them efficient drug-delivery vehicles [36]. Liposomes are lipid vesicles which have been extensively exploited as conventional drug carriers to encapsulate a variety of diagnostic and therapeutic agents with high encapsulation efficiency and protect them from undergoing metabolism [37,38]. Liposomes are able to encapsulate both hydrophilic and hydrophobic drug candidates, the lipid layer of liposomes can accommodate hydrophobic drugs due to hydrophobic interactions between the fatty acid chains and the drug while the aqueous core of the liposome is effective in holding hydrophilic drugs [39]. The major advantage of these liposomes is that they are biocompatible and biodegradable in nature which makes them environmentally friendly [40]. Lipid-based nanocarriers are effectively utilized by pharmaceutical industries for the intracellular delivery of anticancer agents since they exert targeted and controlled drug delivery to cancer cells. Since they exert targeted drug delivery nonspecific activity on the cells, they will reduce which prevents unwanted side effects [41].

**Scheme 1:**
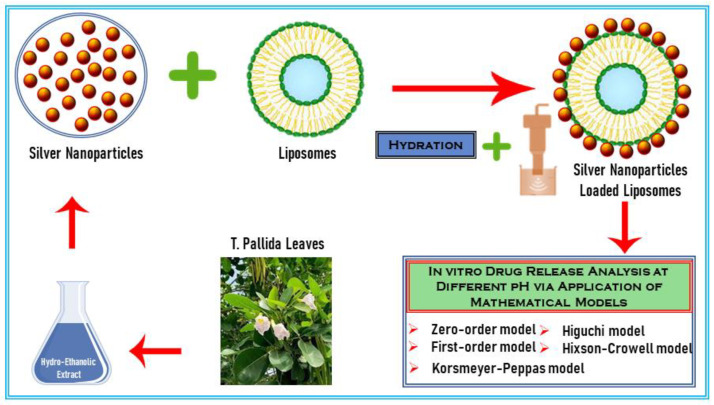
Schematic representation of the green synthesized silver nanoparticle-loaded liposomes and their in vitro drug release analysis using various mathematical models.

Thus, the present study aims to prepare silver nanoparticle-loaded liposome-based nano-system for the purpose of drug delivery. To achieve this, silver nanoparticles were obtained by using *Tabebuia pallida* plant extract as a reducing agent (cf. Scheme 1). During this study, the in vitro drug release profile of the silver-loaded liposome vehicle was evaluated and validated through various mathematical models which could be employed for the treatment of various tumors.

## 2. Materials and Methods

### 2.1. Materials

All the chemicals used in the study were of analytical grade.

### 2.2. Preparation of Tabebuia pallida Leaf Extract

For this study, *Tabebuia pallida* leaves were collected from Avinashi (Tirupur District, Tamilnadu). The identity of the plant (Authentication no: BSI/SRC/5/23/2019/Tech./193) was confirmed by the Botanical Survey of India [BSI], Coimbatore, India. After collection, the leaves of *T. pallida* were washed with tap water several times. Subsequently, the leaves were allowed to dry, which is later transformed into coarsely grinded powder. For the purpose of extraction, 10 g of leaves powder was dispersed in 100 mL of water and ethanol mixture (ethanol: water—60:40). The mixture was hydro distilled under reflux in a Soxhlet apparatus. Later, the residual mixture was filtered using Whatman filter paper and stored in the refrigerator for later use. The freshly prepared hydroethanolic extract was applied for the sunlight-mediated preparation of silver nanoparticles.

### 2.3. Preparation of Silver Nanoparticles

Silver nanoparticles were prepared by using a green synthetic method which is applied in our previous study [26]. Briefly, 10 mL of the as-obtained leaf extract was mixed with 90 mL of freshly prepared silver nitrate solution (1 mM). The final volume of the solution was 100 mL. The subsequent mixture was directly placed under sunlight for several minutes (20 min). Under sunlight, the color of the solution turned from light brown to dark reddish brown which indicated the reduction of silver ions to silver nanoparticles. To isolate the product, the solution was centrifuged at 15,000 rpm for 45 min. After decanting the supernatant, the solid residual silver nanoparticles were taken out and washed several times with DI water to remove the remaining contents of the leaf extract. Later, the as-obtained silver nanoparticles were subjected to lyophilization and stored in the dark for further analysis

### 2.4. Preparation of Silver Nanoparticle-Loaded Liposomes

The silver nanoparticle-encapsulated liposomes were prepared using the thin-film hydration method coupled with sonication. Cholesterol and phosphatidylcholine (Lecithin) were used at a molar ratio of 2:1. The mixture of cholesterol and lecithin was dissolved in 10 mL of chloroform until the formation of a clear solution. Using a rotary evaporator, the chloroform was evaporated at 40 °C and then the flask was kept in a vacuum overnight for the complete removal of the organic solvent which resulted in the formation of a thin lipid film. This thin film was then hydrated with the 5 mL of silver nanoparticles dissolved in DMSO by placing it in a rotary evaporator for 5 min. Thus, obtained silver nanoparticle-loaded liposomes were subjected to sonication to reduce the size of the liposomes. Then, the nonloaded silver nanoparticles present in the supernatant were removed by centrifugation [42].

### 2.5. Encapsulation Efficiency of Silver Nanoparticle-Loaded Liposomes

The silver nanoparticle encapsulation efficiency was determined using the indirect spectrophotometric method. To determine the amount of encapsulated silver nanoparticles, the silver nanoparticle-loaded liposomes were treated with chloroform and shaken well. This process releases the silver nanoparticles encapsulated in the liposomes which can be measured spectrophotometrically at 450 nm. The encapsulation efficiency can be calculated using the following formula [43,44].

Loading capacity = (Amount of encapsulated nanoparticle/weight of the lipid) × 100

$$\mathrm{Encapsulation}\;\mathrm{Efficiency}=\frac{\mathrm{Amount}\;\mathrm{of}\;\mathrm{Encapsulated}\;\mathrm{Nanoparticle}\;}{\mathrm{Amount}\;\mathrm{of}\;\mathrm{Encapsulated}\;+\mathrm{Free}\;\mathrm{Nanoparticle}\;}\times 100$$

### 2.6. Characterization of Silver Nanoparticle-Loaded Liposomes

The particle size distribution (PDI), particle size, and zeta potential of the silver nanoparticle-loaded liposomes were analyzed using DLS (Malvern Instruments, Malvern, UK) method. The size and morphology of the prepared liposomes were analyzed using FESEM (MIRA 3 TESCAN). Initially, the silver nanoparticle-loaded liposomal powder was dissolved in DMSO and subjected to FESEM analysis. The sample was sputtered using gold, dried, and analyzed. To study the interaction between the silver nanoparticles and the lipids the FTIR spectroscopic analysis was carried out (FTIR spectroscopy—miracle 10, SHIMADZU).

### 2.7. In Vitro Drug Release Study

The in vitro release of silver nanoparticles from the liposomes was analyzed using three different pHs: pH 7.4 which corresponds to the pH of blood, pH 6.8 which corresponds to the pH of cancer cells, and pH 5.5 which corresponds to the mature Endosomes of cancer cells. The *Tabebuia pallida* silver nanoparticle-loaded liposomes were transferred to a dialysis bag. The dialysis bag was kept in 100 mL of phosphate-buffered saline at the various pHs 5.5, 6.8, and 7.4 which is kept under a magnetic stirrer for continuous shaking. A total of 5ml of the PBS solution was removed from the system and replaced with 5 mL of fresh PBS solution at various time intervals, namely, 2, 4, 8, 12, 24, 48, and 72 h, to determine the number of silver nanoparticles released from the liposomes. The amount of silver nanoparticles released was estimated spectrophotometrically at 450 nm [45].

### 2.8. Application of Mathematical Models in Release Kinetics of Silver Nanoparticle-Loaded Liposomes

Various mathematical models are employed to correlate the in vitro drug release profile with drug release kinetics. They include the zero-order model, first-order model, the Higuchi model, the Korsmeyer–Peppas model, and the Hixson–Crowell model. To study the Zero-order release kinetics a graph was plotted between cumulative percentage drug release and time. The first-order model can be represented by plotting the log % of drug remaining vs. time. To study the Higuchi model for drug release, a graph was plotted between the cumulative percentage of drug release vs. the square root of time. Log cumulative percentage drug release vs. log time can be used to study the Korsmeyer and Peppas model. To study the Hixson and Crowell model, a graph was plotted between the cube root of the percentage of drug remaining versus time. Various mathematical models can be expressed using the following equations [46].

**Zero-order model**

C_t_ = C_0_ + K_0_ t

where C_t_—the amount of drug released at time t; C_0_—initial concentration of drug at time t = 0; K_0_—zero-order rate constant.

**First-order model**

log C = log C_0_ − K_1_ t/2.303

where K_1_—first-order rate equation expressed in time^−1^ or per hour, C_0_ is the initial concentration of the drug, C is the percent of drug remaining at time t.

**Higuchi model**

Q = K_H_ × t^1/2^

where K_H_—Higuchi dissolution constant; Q—the cumulative amount of drug released in time t per unit area.

**Korsmeyer–Peppas model**

log(M_t_/M_∞_) = log K_kp_ + nlog t

where M_t_—the amount of drug released in time t; M_∞_—the amount of drug released after time ∞; n—the diffusional exponent or drug release exponent; K_kp_—Korsmeyer release rate constant.

**Hixson–Crowell model**

W0^1/3^ − Wt^1/3^ = K_HC_^t^

where W_0_—the initial amount of drug in the liposome; Wt is the remaining amount of drug in the liposome at time t; K_HC_—Hixson–Crowell constant.

## 3. Results and Discussion

**Figure 1:**
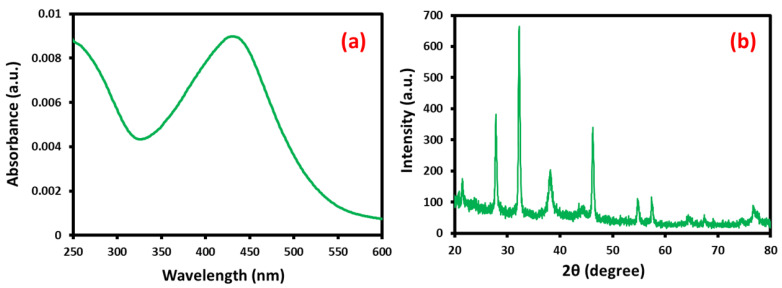
(**a**) UV–visible, and (**b**) XRD analysis of silver nanoparticles.

To prepare the silver nanoparticle-loaded liposomes nano-system, the silver nanoparticles were obtained by using *T. pallida* leaf extract as a reducing agent. The identity of the nanoparticles was confirmed by the UV and XRD analysis. As shown in Figure 1a, the UV spectrum of silver nanoparticles displays a sharp absorption at ~430 nm which is a characteristic peak of silver. In addition, the XRD spectrum in Figure 1b, shows various diffraction peaks at 2θ = 32.20, 38.08, 46.18, and 54.80 corresponding to the (111), (200), (120), and (202) planes, respectively [26]. These diffraction patterns clearly indicate the formation of silver nanoparticles in a face-centered cubic crystalline structure (*fcc*). The size and shape of the silver nanoparticles were confirmed using FESEM analysis and it was found that the synthesized silver nanoparticles were spherical in shape with the size ranging from 10 to 60 nm and the average size was found to be 27 nm.

### 3.1. Encapsulation Efficiency of Silver Nanoparticle-Loaded Liposomes

Encapsulation efficiency is defined as the percentage of drug or nanoparticle that is entrapped successfully by the lipid vesicle. The encapsulation efficiency is calculated using the formula.

$$\mathrm{Encapsulation}\;\mathrm{Efficiency}=\frac{\mathrm{Amount}\;\mathrm{of}\;\mathrm{Encapsulated}\;\mathrm{Nanoparticle}\;}{\mathrm{Amount}\;\mathrm{of}\;\mathrm{Encapsulated}\;+\mathrm{Free}\;\mathrm{Nanoparticle}\;}\times 100$$

**Figure 2:**
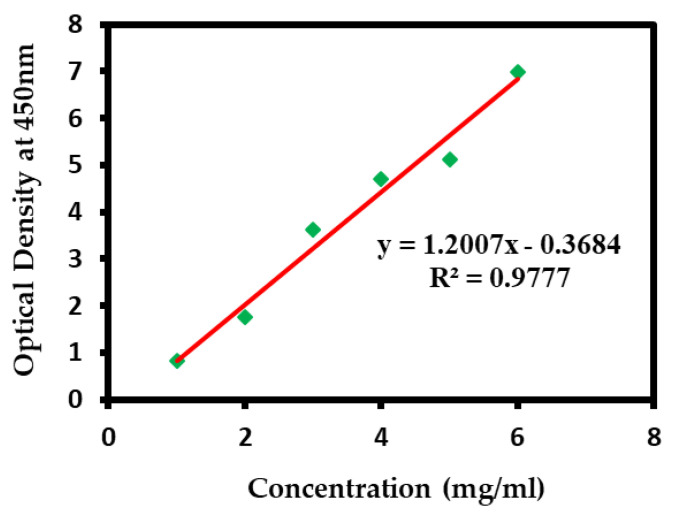
Encapsulation efficiency of silver nanoparticle-loaded liposomes.

From Figure 2, it was found that the amount of encapsulated nanoparticles = 4.1125 mg and the amount of encapsulated + free nanoparticles = 5 mg (amount of nanoparticle present in the liposome initially). By applying these values in the above formula, the encapsulation efficiency was calculated as 82.25%. This high encapsulation efficiency is mainly because the silver nanoparticle is poorly soluble in an aqueous medium which facilitated their incorporation in the hydrophobic part of the liposome. In addition, there is adequate space in the lecithin and cholesterol for the binding of silver nanoparticles because of the suitable ratio between lecithin, cholesterol, and silver nanoparticles, which increased the encapsulation efficiency [44,47]. In line with our study, Hardiansyah et al. 2017 loaded curcumin in both PEGylated magnetic liposomes and found that the PEGylated liposomes possess a higher encapsulation efficiency for curcumin (78.06 ± 0.57%) compared to PEGylated magnetic liposomes (76.15 ± 1.6%) [48]. The curcumin was encapsulated in the lipophilic part of the lipid bilayer of the liposome. Najlah et al. (2019) developed disulfiram encapsulated PEGylated liposome which showed an encapsulation efficiency of more than 80% [49]. The loading capacity of the silver nanoparticle-loaded liposomes has been calculated using the following formula.

Loading capacity = (Amount of encapsulated nanoparticle/weight of the lipid) × 100

From the formula, the loading capacity was found to be 17.74%.

### 3.2. Characterization of Silver Nanoparticle-Loaded Liposomes

#### 3.2.1. Dynamic Light Scattering

An effective drug delivery carrier should possess two main characteristics which include appropriate particle size and surface charge [50,51]. A carrier that fails in these properties will be ineffective and will not exert targeted delivery. Many researchers have reported that when the particle size of the drug carrier increases there will be a decrease in the efficiency of cellular uptake. The optimum size of the drug-loaded carrier is reported as 100–200 nm [52]. This small size can facilitate the drug-loaded carrier to effectively deliver the drug to the targeted tumor site [53,54]. Another important property of the drug delivery carrier is its surface charge which makes them stable under physiological conditions. The high negative charge of the carriers will make them very stable by preventing them from agglomeration and also this negative charge prevents them from being phagocytized to some extent when compared to the positively charged particles [55]. Thus, zeta potential and particle size play a crucial role in drug delivery applications. Usually, a low polydispersity index value of less than 0.5 indicates that the particles are uniformly distributed [56]. This could be attained by subjecting the liposomes to sonication and as a result, the PDI value narrows [57].

**Figure 3:**
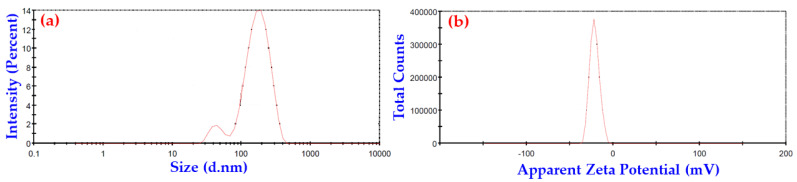
(**a**) Particle size of the silver nanoparticle-loaded liposome, and (**b**) zeta potential of the silver nanoparticle loaded liposome.

As shown in Figure 3a,b, the particle size and zeta potential of the silver nanoparticle-loaded liposomes were found to be 172.1 nm and −21.5 mV, respectively, with the polydispersity index of 0.381. From these results, it is confirmed that the silver nanoparticle-loaded liposomes can effectively target the tumor site because of their size of less than 200 nm and will not agglomerate easily, which increases its stability. In line with our study, Joel et al. prepared liposomes loaded with silver nanoparticles obtained by green synthesis and observed that the polydispersity index, average size, and zeta potential were 0.384, 129.12−278.46 nm, and −18.96 mV, respectively [58].

#### 3.2.2. Field Emission Scanning Electron Microscope

**Figure 4:**
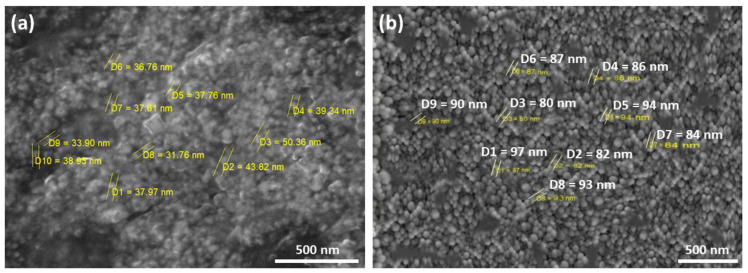
FESEM image of (**a**) silver nanoparticles and (**b**) silver nanoparticle-loaded liposomes.

The size and morphology of the silver nanoparticle-loaded liposomes were analyzed using a field emission scanning electron microscope (FESEM). FESEM analysis revealed the presence of spherical shape AgNP-loaded liposomes which are in the size range of 80–97 nm (Figure 4). The size of the silver nanoparticle-loaded liposomes that are observed in SEM analysis is lesser than the size observed in DLS analysis. This is because the SEM measures the number-based size distribution and it will not measure the ions and molecules that are present on the surface of the particle. However, DLS is based on the measurement of hydrodynamic size which includes capping agent. Our results coincide with the study conducted by Yusuf et al., in which the silver nanoparticles were uniformly distributed, and spherical in shape with ~82 nm in size in the composite of encapsulated silver nanoparticles with liposomes [35].

#### 3.2.3. Fourier Transform Infrared Spectroscopy

**Figure 5:**
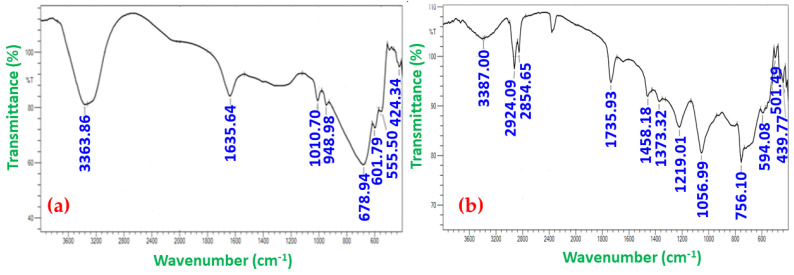
(**a**) FTIR spectrum of blank liposomes (lecithin and cholesterol), and (**b**) FTIR spectrum of silver nanoparticle-loaded liposomes.

The FTIR spectral analysis was carried out to analyze the functional groups that play a role in the formation of silver nanoparticle-loaded liposomes. The FTIR spectrum was recorded for both the blank liposomes (lecithin and cholesterol alone- before the encapsulation of silver nanoparticles) and the silver nanoparticle-loaded liposomes (Figure 5a,b). The FTIR spectrum of the blank liposomes shows various peaks at different wave numbers including 555, 601, 678, 948, 1010, 1635, and 3363 cm^−1^. These peaks correspond to various functional groups which are as follows: a peak at 555 cm^−1^ represents C-Br stretching which indicates the presence of halo compounds, and another band at 601 cm^−1^ corresponds to the C-Cl which indicates alkyl halides. A peak at 678 cm^−1^ corresponds to =CH which indicates aromatic compounds [59]. A peak at 1010 cm^−1^ and 948 cm^−1^ represents C=C, respectively. FTIR spectrum at 1635 cm^−1^ and 3363 cm^−1^ corresponds to C=O and O-H bonds respectively [60].

The FTIR spectrum of silver nanoparticle-loaded liposomes shows different peaks at 501, 594, 756, 1056, 1219, 1373, 1458, 1735, 2854, 2924, and 3387 cm^−1^. A peak at 501 cm^−1^ and 594 cm^−1^ corresponds to C-I. An FTIR peak at 756 cm^−1^ corresponds to the C=C group [24]. A peak at 1056 cm^−1^ represents the C-O bond and a peak at 1219 cm^−1^ represents -CO-. FTIR peak at 1373 cm^−1^ and 1458 cm^−1^ corresponds to -CH_2_ bond respectively. A peak at 1735 cm^−1^ corresponds to the -C-O-C- group. FTIR peak at 2854 cm^−1^ and 2924 cm^−1^ corresponds to the -CH _3_ group. Finally, a peak at 3387 cm^−1^ represents the O-H bond. From the FTIR spectrum of liposome blank and silver nanoparticle-loaded liposomes, it is observed that there is an interaction between the silver nanoparticles and the lipids which leads to their encapsulation inside the vesicles [61].

### 3.3. In Vitro Drug Release Study

**Figure 6:**
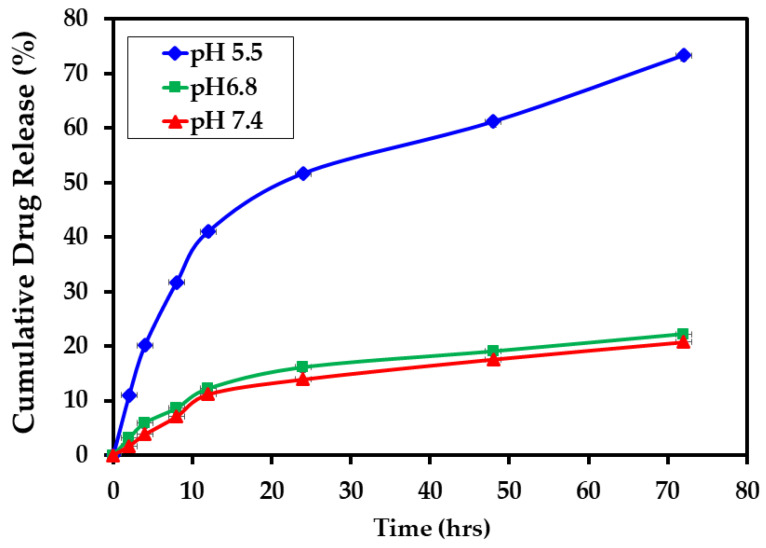
In vitro drug release profile of silver nanoparticle-loaded liposomes (the values are represented as ±SD for triplicates in each category).

The in vitro release of silver nanoparticles from the liposomes were analyzed using three different pH; pH 7.4 which corresponds to the pH of blood, pH 6.8 which corresponds to the pH of cancer cells and pH 5.5 which corresponds to the mature Endosomes of cancer cells for different time intervals such as 2, 4, 8, 12, 24, 48, and 72 h. The in vitro drug release profile of silver nanoparticle-loaded liposomes are shown in Figure 6.

From the graph, a high silver nanoparticle release from liposomes was observed at pH 5.5 which corresponds to the mature Endosomes of cancer cells. The drug release was found to be increased when the time increased in all the pH models: 73.32 ± 0.68% nanoparticles were released in the system after 72 h at a pH of 5.5, and 22.24 ± 1.00% nanoparticles and 20.75 ± 0.11% nanoparticles were released at the 72 h at a pH of 6.8 and 7.4, respectively. A high silver nanoparticle release was observed at low pH and the drug release was found to be decreased when the pH of the system increased. From this evidence, it was confirmed that the drug release was high in acidic conditions. This is because the acidic condition breaks the liposomal integrity and releases the encapsulated silver nanoparticle. This acidic condition promotes the hydrolysis of phospholipids which increases membrane permeability and leads to the release of the encapsulated nanoparticles. However, the liposome structure is resistant to alkali conditions [62]. Similar findings were indicated by Ankita et al. (2020) who synthesized Paclitaxel and Piperine co-loaded liposomes and carried out an in vitro drug release study by the dialysis bag method. They analyzed the release of Paclitaxel and Piperine in three different pHs: pH 5.5, pH 6.5, and pH 7.4 and observed that there is a high release of the encapsulated drug in pH 5.5 [63].

### 3.4. Application of Mathematical Models in Release Kinetics of Silver Nanoparticle-Loaded Liposomes

#### 3.4.1. Zero-Order Model

**Figure 7:**
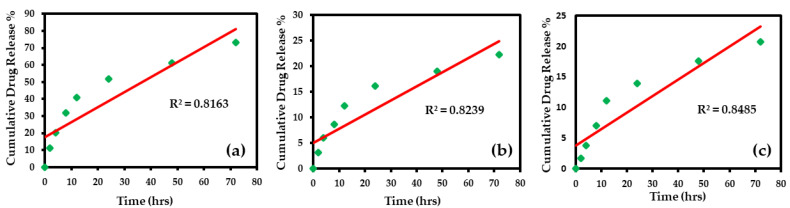
(**a**) Zero-order release kinetics for pH 5.5, (**b**) zero-order release kinetics for pH 6.8, and (**c**) zero-order release kinetics for pH 7.4.

According to zero-order drug release kinetics, a drug release from a drug delivery system remains constant per unit of time and it is independent of concentration. To evaluate the drug release by zero-order kinetics, a graph was plotted between time and cumulative drug release % (Figure 7a–c). The slope of the graph provides a rate constant for the zero-order model. The correlation coefficient R^2^ can be used to predict whether the drug release follows a zero-order model or not [64].

From the above graphs, it could be concluded that the drug release does not follow zero-order release kinetics as there is a lower value (R^2^ = 0.816 for pH 5.5, R^2^ = 0.823 for pH 6.8, and R^2^ = 0.848 for pH 7.4) of the correlation coefficient. 

#### 3.4.2. First-Order Model

**Figure 8:**
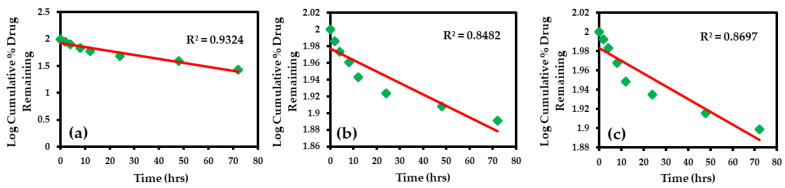
(**a**) First order release kinetics for pH 5.5, (**b**) first order release kinetics for pH 6.8, and (**c**) first order release kinetics for pH 7.4.

According to first-order drug release kinetics, the drug release rate is proportional to the drug concentration which indicates that when there is a high concentration of the drug, the release rate also will be high and when the drug concentration decreases, the drug release also decreases [64]. To study the first-order release kinetics for various pHs, a graph was plotted between the log % of drug remaining and time (Figure 8a–c). From the slope of the line, the first-order rate constant can be predicted and the correlation coefficient value indicates whether the drug release obeys first-order kinetics or not. 

From the above graphs, it could be concluded that the drug release does not follow First order release kinetics as there is a lower value (R^2^ = 0.932 for pH 5.5, R^2^ = 0.848 for pH 6.8, and R^2^ = 0.869 for pH 7.4) of the correlation coefficient. 

#### 3.4.3. Higuchi Model

**Figure 9:**
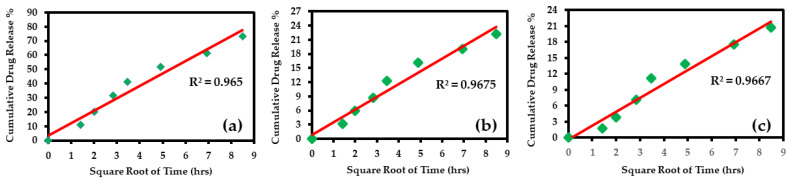
(**a**) Higuchi drug release kinetics for pH 5.5, (**b**) Higuchi drug release kinetics for pH 6.8, and (**c**) Higuchi drug release kinetics for pH 7.4.

According to the Higuchi model, the drug release from a matrix as a square root of time is based on the process of diffusion. If there is the highest correlation coefficient, then it can be predicted that the drug release is through diffusion. There are some predictions for the Higuchi model and they include: the drug concentration is much higher than the solubility of the matrix at time t_0_, Sink conditions are maintained perfectly, and there is constant drug diffusion [46]. To study the Higuchi model of drug release kinetics for various pHs, a graph was plotted between cumulative % drug release and square root time (Figure 9a–c).

From the above graphs, it could be concluded that the drug release perfectly follows Higuchi drug release kinetics as there is the highest value (R^2^ = 0.965 for pH 5.5, R^2^ = 0.967 for pH 6.8, and R^2^ = 0.966 for pH 7.4) of the correlation coefficient. 

#### 3.4.4. Korsmeyer–Peppas Model

**Figure 10:**
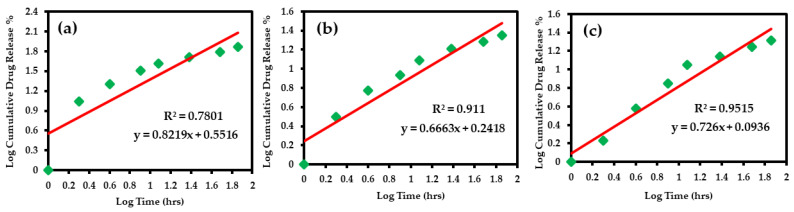
(**a**) Korsmeyer–Peppas drug release kinetics for pH 5.5, (**b**) Korsmeyer–Peppas drug release kinetics for pH 6.8, and (**c**) Korsmeyer–Peppas drug release kinetics for pH 7.4.

From the Higuchi model of drug release kinetics, it is confirmed that the drug release is through diffusion. The Korsmeyer–Peppas model is fitted to understand that drug release follows type of diffusion [65]. To study the drug release kinetics by the Korsmeyer–Peppas model, a graph was plotted between log cumulative drug release % and log time (Figure 10a–c). From the n value (release exponent) obtained using the plot, the type of diffusion can be predicted.

The drug transport mechanism for different values of release exponent was expressed in Table 1. From Table 2, it could be concluded that the drug release of various pH values follows non-Fickian transport as their release exponent falls in the category of 0.5 < n < 1.0 [66].

#### 3.4.5. Hixson–Crowell Model

**Figure 11:**
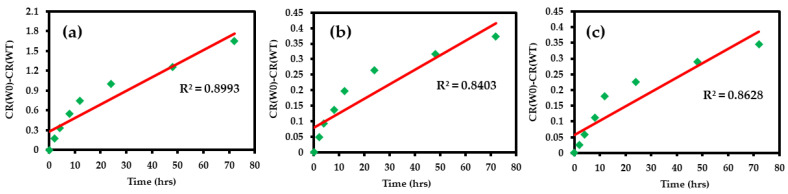
(**a**) Hixson–Crowell drug release kinetics for pH 5.5, (**b**) Hixson–Crowell drug release kinetics for pH 6.8, and (**c**) Hixson–Crowell drug release kinetics for pH 7.4.

According to the Hixson–Crowell model, the drug release is by dissolution and not by diffusion. It is based on the fact that there is a change in the surface area and diameter of the particle when there is dissolution [65]. To study the drug release kinetics by Hixson–Crowell model, a graph was plotted between the cube root of the initial drug concentration and the concentration of the drug at time t against time (Figure 11a–c).

From the above graphs, it could be concluded that the drug release does not follow Hixson–Crowell release kinetics as there is a lower value (R^2^ = 0.899 for pH 5.5, R^2^ = 0.840 for pH 6.8 and, R^2^ = 0.862 for pH 7.4) of the correlation coefficient.

### 3.5. Results of Various Mathematical Models

The in vitro drug release profile of silver nanoparticle-loaded liposomes was fitted for five different mathematical models and evaluated using correlation coefficient r^2^. The results of all five models are given in Table 3. A suitable mathematical model for in vitro drug release study was determined by the highest value of the correlation coefficient. Among all the models analyzed, the Higuchi model was found to be the best-fitted model as it shows the highest degree of correlation coefficient so it could be concluded that the drug release follows the diffusion mechanism. From the Korsmeyer–Peppas model, it was confirmed that the drug release is based on anomalous non-Fickian transport. In line with our study, Nayer et al. prepared turmeric extract-loaded nanoliposomes and carried out an in vitro drug release study and validated the results through various mathematical models, and observed that the Higuchi model was found to be the best-fitted model as its coefficient of correlation is higher when compared to the other models [44]. Similar findings were observed by Minh et al. (2020) who prepared Letrozole and Paclitaxel-loaded liposomes and applied the drug release profile in various mathematical models such as the zero-order model, first-order model, Higuchi model, and Korsmeyer–Peppas model, and observed that the Korsmeyer–Peppas and Higuchi models indicated the highest correlation coefficient values. Since the release profile of Letrozole and Paclitaxel shows the highest correlation coefficient for the Higuchi model, it is confirmed that the drug release follows diffusion, and also in the Korsmeyer and Peppas model, the value of release exponent (n) was found to be lower than 0.5 for both Letrozole and Paclitaxel which indicates that the drug release follows Quasi-Fickian diffusion [67]. Fateme et al. 2018 prepared PEGylated doxorubicin-loaded liposomes and carried out mathematical modeling of drug release kinetics. They observed that the drug release follows the Korsmeyer and Peppas model of drug release kinetics [68]. Milad et al. 2012 prepared hesperetin loaded liposomes and analyzed the in vitro drug release profiling of the liposomes, and validated them through different mathematical models. Among the various mathematical models analyzed, the release of hesperetin from the liposomes was found to adhere to the Rigter–Peppas model of drug release as it has the highest value of correlation coefficient [69].

In agreement with the above-cited literature and the results obtained from our study, it could be concluded that the release of silver nanoparticles from the liposomes follows the Higuchi model of drug release as there is the highest value of correlation coefficient. As the drug release is fitted to the Higuchi model, it could be considered that the drug release is through diffusion. From the results of the Korsmeyer–Peppas model, it is considered that the drug release is based on anomalous non-Fickian transport.

## 4. Conclusions

Herein, we have demonstrated the in vitro drug release analysis of the silver nanoparticle-loaded liposome hybrids, which was performed by using the dialysis bag method. To prepare the hybrids, pristine silver nanoparticles were synthesized using an eco-friendly plant extract-based method in which *T. pallida* leaf extract was used as a reducing agent. On the other hand, the silver nanoparticle and liposome hybrids were prepared by using the thin-film hydration method coupled with sonication. Detailed analysis of the sample has revealed that the prepared silver nanoparticle-encapsulated liposome hybrids were highly stable, and polydisperse with less aggregation of nanoparticles. These hybrids exerted targeted delivery of bioactive silver to the cancer cells because of their smaller size. Furthermore, the in vitro drug release studies validated the controlled and targeted delivery of silver nanoparticles by liposomes to the cancer cells. To further validate, the in vitro drug release profile was also fitted in five different mathematical models and evaluated using correlation coefficient r^2^. The mathematical models of drug release kinetics have revealed that the release exponent (n) of the samples at different pH was recorded between 0.66 to 0.82, which strongly points towards a drug release mechanism that follows a non-Fickian diffusion method. This type of drug release mechanism is typically followed by the majority of drug delivery systems comprising liposomes. Particularly, the Korsmeyer–Peppas model used in this study has been established as the most effective model to evaluate liposome-based drug delivery systems, which provide crucial information on the release mechanism from the carrier. Moreover, this method requires fewer controls, unlike the zero-order approximation.

## Figures and Tables

**Table 1:** The drug transport mechanism for different values of release exponent (n).

Release Exponent (n)	Drug Transport Mechanism
n < 0.5	Quasi-Fickian diffusion
0.5	Fickian diffusion
0.5 < n < 1. 0	Anomalous (Non-Fickian transport)
1.0	Case II transport
Higher than1.0	Super case II transport

**Table 2:** Drug transport mechanism for various pH (pH 5.5, pH 6.8, and pH 7.4).

pH	Equation	Release Exponent (n)	Drug Transport Mechanism
5.5	y = 0.821x + 0.551	0.821	Non-Fickian Transport
6.8	y = 0.666x + 0.241	0.666	
7.4	y = 0.726x + 0.093	0.726	

**Table 3:** Results of Various mathematical models in terms of r^2^, slope, and intercept.

Name of the Mathematical Model	pH 5.5 (Mature Endosomes of Cancer Cells)				pH 6.8 (Cancer Cells)				pH 7.4 (Blood)			
	R^2^	Equation	Slope	Intercept	R^2^	Equation	Slope	Intercept	R^2^	Equation	slope	Intercept
**Zero-order**	0.816	y = 0.884x + 17.47	0.8845	17.474	0.823	y = 0.276x + 5.06	0.2762	5.065	0.848	y = 0.270x + 3.754	0.2705	3.755
**First-order**	0.932	y = −0.007x + 1.92	−0.0072	1.923798	0.848	y = −0.001x + 1.97	−0.0013	1.977	0.869	y = −0.001x + 1.98	−0.0013	1.983
**Higuchi**	0.965	y = 8.687x + 3.673	8.6876	3.673744	0.967	y = 2.703x + 0.789	2.7038	0.789104	0.966	y = 2.608x − 0.283	2.6087	−0.28379
**Korsmeyer–Peppas**	0.780	y = 0.821x + 0.551	0.8219	0.551602	0.911	y = 0.666x + 0.241	0.6663	0.241776	0.951	y = 0.726x + 0.093	0.7259	0.093642
**Hixson–Crowell**	0.899	y = 0.020x + 0.276	0.0206	0.276325	0.840	y = 0.004x + 0.079	0.0046	0.079622	0.862	y = 0.004x + 0.058	0.0045	0.058526

## Data Availability

Data are contained within the article.

## References

[1] Bray F., Ferlay J., Soerjomataram I., Siegel R.L., Torre L.A., Jemal A. (2018). Global cancer statistics 2018: GLOBOCAN estimates of incidence and mortality worldwide for 36 cancers in 185 countries. CA: A Cancer J. Clin..

[2] Sung H., Ferlay J., Siegel R.L., Laversanne M., Soerjomataram I., Jemal A., Bray F. (2021). Global cancer statistics 2020: GLOBOCAN estimates of incidence and mortality worldwide for 36 cancers in 185 countries. CA: A Cancer J. Clin..

[3] Hassanpour S.H., Dehghani M. (2017). Review of cancer from perspective of molecular. J. Cancer Res. Pract..

[4] Khan M., Khan M., Al-Marri A.H., Al-Warthan A., Alkhathlan H.Z., Siddiqui M.R.H., Nayak V.L., Kamal A., Adil S.F. (2016). Apoptosis inducing ability of silver decorated highly reduced graphene oxide nanocomposites in A549 lung cancer. Int. J. Nanomed..

[5] Dotto G.P., Rustgi A.K. (2016). Squamous cell cancers: A unified perspective on biology and genetics. Cancer Cell.

[6] National Cancer Institute, USA. https://www.cancer.gov/about-cancer/understanding/what-is-cancer.

[7] Crafton S.M., Salani R. (2016). Beyond chemotherapy: An overview and review of targeted therapy in cervical cancer. Clin. Ther..

[8] Schirrmacher V. (2019). From chemotherapy to biological therapy: A review of novel concepts to reduce the side effects of systemic cancer treatment. Int. J. Oncol..

[9] Yao Y., Ji C., He Y., Pan Y. (2017). Relationship between Helicobacter pylori infection and vomiting induced by gastrointestinal cancer chemotherapy. Intern. Med. J..

[10] Altun I., Sonkaya A. (2018). The most common side effects experienced by patients were receiving first cycle of chemotherapy. Iran. J. Public Health.

[11] Asimuddin M., Shaik M.R., Adil S.F., Siddiqui M.R.H., Alwarthan A., Jamil K., Khan M. (2020). Azadirachta indica based biosynthesis of silver nanoparticles and evaluation of their antibacterial and cytotoxic effects. J. King Saud Univ. Sci..

[12] Prasher P., Sharma M., Mudila H., Gupta G., Sharma A.K., Kumar D., Bakshi H.A., Negi P., Kapoor D.N., Chellappan D.K. (2020). Emerging trends in clinical implications of bio-conjugated silver nanoparticles in drug delivery. Colloid Interface Sci. Commun..

[13] Khan M., Al-Hamoud K., Liaqat Z., Shaik M.R., Adil S.F., Kuniyil M., Alkhathlan H.Z., Al-Warthan A., Siddiqui M.R.H., Mondeshki M. (2020). Synthesis of Au, Ag, and Au–Ag Bimetallic Nanoparticles Using Pulicaria undulata Extract and Their Catalytic Activity for the Reduction of 4-Nitrophenol. Nanomaterials.

[14] Roy A., Bulut O., Some S., Mandal A.K., Yilmaz M.D. (2019). Green synthesis of silver nanoparticles: Biomolecule-nanoparticle organizations targeting antimicrobial activity. RSC Adv..

[15] Ahmad S., Munir S., Zeb N., Ullah A., Khan B., Ali J., Bilal M., Omer M., Alamzeb M., Salman S.M. (2019). Green nanotechnology: A review on green synthesis of silver nanoparticles—An ecofriendly approach. Int. J. Nanomed..

[16] Moradi F., Sedaghat S., Moradi O., Arab Salmanabadi S. (2021). Review on green nano-biosynthesis of silver nanoparticles and their biological activities: With an emphasis on medicinal plants. Inorg. Nano-Met. Chem..

[17] Garg D., Sarkar A., Chand P., Bansal P., Gola D., Sharma S., Khantwal S., Mehrotra R., Chauhan N., Bharti R.K. (2020). Synthesis of silver nanoparticles utilizing various biological systems: Mechanisms and applications—A review. Prog. Biomater..

[18] Ratan Z.A., Haidere M.F., Nurunnabi M., Shahriar S.M., Ahammad A.S., Shim Y.Y., Reaney M.J., Cho J.Y. (2020). Green chemistry synthesis of silver nanoparticles and their potential anticancer effects. Cancers.

[19] Israel L.L., Galstyan A., Holler E., Ljubimova J.Y. (2020). Magnetic iron oxide nanoparticles for imaging, targeting and treatment of primary and metastatic tumors of the brain. J. Control. Release.

[20] Ali M.A., Ahmed T., Wu W., Hossain A., Hafeez R., Islam Masum M.M., Wang Y., An Q., Sun G., Li B. (2020). Advancements in plant and microbe-based synthesis of metallic nanoparticles and their antimicrobial activity against plant pathogens. Nanomaterials.

[21] Khan M., Shaik M.R., Adil S.F., Khan S.T., Al-Warthan A., Siddiqui M.R.H., Tahir M.N., Tremel W. (2018). Plant extracts as green reductants for the synthesis of silver nanoparticles: Lessons from chemical synthesis. Dalton Trans..

[22] Rahman M., Hossain A., Mostofa M., Khan M.A., Ali R., Mosaddik A., Sadik M., Alam A. (2019). Evaluation of anti-ROS and anticancer properties of *Tabebuia pallida* L. Leaves. Clin. Phytoscience.

[23] El-Hawary S.S., Mohammed R., Tawfike A.F., AbouZid S.F., Taher M.A., Abdelmohsen U.R., Amin E. (2021). Metabolic profiling of cytotoxic metabolites from five Tabebuia species supported by molecular correlation analysis. Sci. Rep..

[24] Ferraz-Filha Z.S., Ferrari F.C., Araújo M.C.d.P.M., Bernardes A.C.F.P. (2017). Effects of the aqueous extract from Tabebuia roseoalba and phenolic acids on hyperuricemia and inflammation. Evid. -Based Complement. Altern. Med..

[25] Regalado A.I., Mancebo B., Paixão A., López Y., Merino N., Sánchez L.M. (2017). Antinociceptive activity of methanol extract of Tabebuia hypoleuca (C. Wright ex Sauvalle) Urb. stems. Med. Princ. Pract..

[26] Jayachandran P., Ilango S., Nirmaladevi R. (2021). Evaluation of antioxidant potential of green synthesized tabebuia pallida silver nanoparticles. J. Adv. Sci. Res..

[27] Mbanga O., Cukrowska E., Gulumian M. (2022). Dissolution kinetics of silver nanoparticles: Behaviour in simulated biological fluids and synthetic environmental media. Toxicol. Rep..

[28] Prasher P., Singh M., Mudila H. (2018). Oligodynamic effect of silver nanoparticles: A review. BioNanoScience.

[29] Prasher P., Sharma M. (2021). Antimicrobial properties of surface-functionalized silver nanoparticles. Silver Nanomaterials for Agri-Food Applications.

[30] Ravindran A., Chandran P., Khan S.S. (2013). Biofunctionalized silver nanoparticles: Advances and prospects. Colloids Surf. B Biointerfaces.

[31] Sarkar K., Banerjee S.L., Kundu P.P., Madras G., Chatterjee K. (2015). Biofunctionalized surface-modified silver nanoparticles for gene delivery. J. Mater. Chem. B.

[32] Rezazadeh N.H., Buazar F., Matroodi S. (2020). Synergistic effects of combinatorial chitosan and polyphenol biomolecules on enhanced antibacterial activity of biofunctionalized silver nanoparticles. Sci. Rep..

[33] Xue Y., Zhang S., Huang Y., Zhang T., Liu X., Hu Y., Zhang Z., Tang M. (2012). Acute toxic effects and gender-related biokinetics of silver nanoparticles following an intravenous injection in mice. J. Appl. Toxicol..

[34] Barbinta-Patrascu M.E., Ungureanu C., Iordache S.M., Bunghez I.R., Badea N., Rau I. (2014). Green silver nanobioarchitectures with amplified antioxidant and antimicrobial properties. J. Mater. Chem. B.

[35] Yusuf A., Brophy A., Gorey B., Casey A. (2018). Liposomal encapsulation of silver nanoparticles enhances cytotoxicity and causes induction of reactive oxygen species-independent apoptosis. J. Appl. Toxicol..

[36] Guimarães D., Cavaco-Paulo A., Nogueira E. (2021). Design of liposomes as drug delivery system for therapeutic applications. Int. J. Pharm..

[37] Riaz M.K., Riaz M.A., Zhang X., Lin C., Wong K.H., Chen X., Zhang G., Lu A., Yang Z. (2018). Surface functionalization and targeting strategies of liposomes in solid tumor therapy: A review. Int. J. Mol. Sci..

[38] Shivanna A.T., Dash B.S., Chen J.-P. (2022). Functionalized Magnetic Nanoparticles for Alternating Magnetic Field-or Near Infrared Light-Induced Cancer Therapies. Micromachines.

[39] Large D.E., Abdelmessih R.G., Fink E.A., Auguste D.T. (2021). Liposome composition in drug delivery design, synthesis, characterization, and clinical application. Adv. Drug Deliv. Rev..

[40] TS A., Shalumon K., Chen J.-P. (2019). Applications of magnetic liposomes in cancer therapies. Curr. Pharm. Des..

[41] Bozzuto G., Molinari A. (2015). Liposomes as nanomedical devices. Int. J. Nanomed..

[42] Fathy M.M., Fahmy H.M., Balah A.M., Mohamed F.F., Elshemey W.M. (2019). Magnetic nanoparticles-loaded liposomes as a novel treatment agent for iron deficiency anemia: In vivo study. Life Sci..

[43] Lu Y.-J., Chuang E.-Y., Cheng Y.-H., Anilkumar T., Chen H.-A., Chen J.-P. (2019). Thermosensitive magnetic liposomes for alternating magnetic field-inducible drug delivery in dual targeted brain tumor chemotherapy. Chem. Eng. J..

[44] Karimi N., Ghanbarzadeh B., Hajibonabi F., Hojabri Z., Ganbarov K., Kafil H.S., Hamishehkar H., Yousefi M., Mokarram R.R., Kamounah F.S. (2019). Turmeric extract loaded nanoliposome as a potential antioxidant and antimicrobial nanocarrier for food applications. Food Biosci..

[45] Kavithaa K., Paulpandi M., Padma P.R., Sumathi S. (2016). Induction of intrinsic apoptotic pathway and cell cycle arrest via baicalein loaded iron oxide nanoparticles as a competent nano-mediated system for triple negative breast cancer therapy. RSC Adv..

[46] Gouda R., Baishya H., Qing Z. (2017). Application of mathematical models in drug release kinetics of carbidopa and levodopa ER tablets. J. Dev. Drugs.

[47] TS A., Lu Y.-J., Chen J.-P. (2020). Optimization of the preparation of magnetic liposomes for the combined use of magnetic hyperthermia and photothermia in dual magneto-photothermal cancer therapy. Int. J. Mol. Sci..

[48] Hardiansyah A., Yang M.-C., Liu T.-Y., Kuo C.-Y., Huang L.-Y., Chan T.-Y. (2017). Hydrophobic drug-loaded PEGylated magnetic liposomes for drug-controlled release. Nanoscale Res. Lett..

[49] Najlah M., Said Suliman A., Tolaymat I., Kurusamy S., Kannappan V., Elhissi A.M., Wang W. (2019). Development of injectable PEGylated liposome encapsulating disulfiram for colorectal cancer treatment. Pharmaceutics.

[50] Yoshizawa Y., Kono Y., Ogawara K.-i., Kimura T., Higaki K. (2011). PEG liposomalization of paclitaxel improved its in vivo disposition and anti-tumor efficacy. Int. J. Pharm..

[51] Nguyen Thi P.P., Nguyen M.T., Hai N.D. (2016). Role of collagen concentration in stability of Star-shaped Silver@ Gold nanoparticles. Proceedings of Journal of Nano Research.

[52] Petros R.A., DeSimone J.M. (2010). Strategies in the design of nanoparticles for therapeutic applications. Nat. Rev. Drug Discov..

[53] Aslan B., Ozpolat B., Sood A.K., Lopez-Berestein G. (2013). Nanotechnology in cancer therapy. J. Drug Target..

[54] Nguyen D.H., Lee J.S., Bae J.W., Choi J.H., Lee Y., Son J.Y., Park K.D. (2015). Targeted doxorubicin nanotherapy strongly suppressing growth of multidrug resistant tumor in mice. Int. J. Pharm..

[55] Sheng Y., Chang L., Kuang T., Hu J. (2016). PEG/heparin-decorated lipid–polymer hybrid nanoparticles for long-circulating drug delivery. RSC Adv..

[56] Li Q., Li Z., Zeng W., Ge S., Lu H., Wu C., Ge L., Liang D., Xu Y. (2014). Proniosome-derived niosomes for tacrolimus topical ocular delivery: In vitro cornea permeation, ocular irritation, and in vivo anti-allograft rejection. Eur. J. Pharm. Sci..

[57] Paini M., Daly S.R., Aliakbarian B., Fathi A., Tehrany E.A., Perego P., Dehghani F., Valtchev P. (2015). An efficient liposome based method for antioxidants encapsulation. Colloids Surf. B Biointerfaces.

[58] Espinoza J.T., Novak R.S., Magalhães C.G., Budel J.M., Justus B., Gonçalves M.M., Boscardin P.M.D., Farago P.V., Paula J.d.F.P.D. (2021). Preparation and characterization of liposomes loaded with silver nanoparticles obtained by green synthesis. Braz. J. Pharm. Sci..

[59] Ajitha B., Reddy Y.A.K., Reddy P.S. (2014). Biogenic nano-scale silver particles by Tephrosia purpurea leaf extract and their inborn antimicrobial activity. Spectrochim. Acta Part A Mol. Biomol. Spectrosc..

[60] Sadhasivam L., Durairaj J. (2014). Evaluation profile of silver nanoparticle synthesized by aloe vera extract. Int. J. ChemTech. Res..

[61] Zhang Y., Cheng X., Zhang Y., Xue X., Fu Y. (2013). Biosynthesis of silver nanoparticles at room temperature using aqueous aloe leaf extract and antibacterial properties. Colloids Surf. A Physicochem. Eng. Asp..

[62] Jin H.-H., Lu Q., Jiang J.-G. (2016). Curcumin liposomes prepared with milk fat globule membrane phospholipids and soybean lecithin. J. Dairy Sci..

[63] Burande A.S., Viswanadh M.K., Jha A., Mehata A.K., Shaik A., Agrawal N., Poddar S., Mahto S.K., Muthu M.S. (2020). EGFR targeted paclitaxel and piperine co-loaded liposomes for the treatment of triple negative breast cancer. AAPS PharmSciTech.

[64] Dash S., Murthy P.N., Nath L., Chowdhury P. (2010). Kinetic modeling on drug release from controlled drug delivery systems. Acta Pol. Pharm..

[65] Singhvi G., Singh M. (2011). In-vitro drug release characterization models. Int. J. Pharm. Stud. Res..

[66] Paarakh M.P., Jose P.A., Setty C., Christoper G. (2018). Release kinetics–concepts and applications. Int. J. Pharm. Res. Technol..

[67] Vu M.T., Le N.T.T., Pham T.L.-B., Nguyen N.H., Nguyen D.H. (2020). Development and characterization of soy lecithin liposome as potential drug carrier systems for codelivery of letrozole and paclitaxel. J. Nanomater..

[68] Haghiralsadat F., Amoabediny G., Helder M.N., Naderinezhad S., Sheikhha M.H., Forouzanfar T., Zandieh-Doulabi B. (2018). A comprehensive mathematical model of drug release kinetics from nano-liposomes, derived from optimization studies of cationic PEGylated liposomal doxorubicin formulations for drug-gene delivery. Artif. Cells Nanomed. Biotechnol..

[69] Fathi M., Varshosaz J., Mohebbi M., Shahidi F. (2013). Hesperetin-loaded solid lipid nanoparticles and nanostructure lipid carriers for food fortification: Preparation, characterization, and modeling. Food Bioprocess Technol..

